# Supplementary material for: A Critical View of the Application of the APEX Software (Aqueous Photochemistry of Environmentally-Occurring Xenobiotics) to Predict Photoreaction Kinetics in Surface Freshwaters
Source: Molecules. 2019 Dec 18;25(1):9. doi: 10.3390/molecules25010009 (PMC7017383; doi:10.3390/molecules25010009)
Supplement: Supplementary file 1 [file molecules-25-00009-s001.zip › 3.2.4_gcc-4.4.0.zip!/3.2.4_gcc-4.4.0/3.2.4_gcc-4.4.0/doc/octave/html/interpreter/Delaunay-Triangulation.html]

Delaunay Triangulation - Untitled


Next: Voronoi Diagrams,
Up: Geometry


---

### 29.1 Delaunay Triangulation

The Delaunay triangulation is constructed from a set of
circum-circles. These circum-circles are chosen so that there are at
least three of the points in the set to triangulation on the
circumference of the circum-circle. None of the points in the set of
points falls within any of the circum-circles.

In general there are only three points on the circumference of any
circum-circle. However, in some cases, and in particular for the
case of a regular grid, 4 or more points can be on a single
circum-circle. In this case the Delaunay triangulation is not unique.

— Function File: tri = **delaunay** (x, y)  
— Function File: tri = **delaunay** (x, y, opt)  
> The return matrix of size [n, 3] contains a set triangles which are
> described by the indices to the data point x and y vector.
> The triangulation satisfies the Delaunay circum-circle criterion.
> No other data point is in the circum-circle of the defining triangle.
>
> A third optional argument, which must be a string, contains extra options
> passed to the underlying qhull command. See the documentation for the
> Qhull library for details.
>
> ```
>           x = rand (1, 10);
>           y = rand (size (x));
>           T = delaunay (x, y);
>           X = [x(T(:,1)); x(T(:,2)); x(T(:,3)); x(T(:,1))];
>           Y = [y(T(:,1)); y(T(:,2)); y(T(:,3)); y(T(:,1))];
>           axis ([0,1,0,1]);
>           plot (X, Y, "b", x, y, "r*");
> ```
>
>
>
>
> **See also:** voronoi, delaunay3, delaunayn.

The 3- and N-dimensional extension of the Delaunay triangulation are
given by `delaunay3` and `delaunayn` respectively.
`delaunay3` returns a set of tetrahedra that satisfy the
Delaunay circum-circle criteria. Similarly, `delaunayn` returns the
N-dimensional simplex satisfying the Delaunay circum-circle criteria.
The N-dimensional extension of a triangulation is called a tessellation.

— Function File: T = **delaunay3** (x, y, z)  
— Function File: T = **delaunay3** (x, y, z, opt)  
> A matrix of size [n, 4] is returned. Each row contains a
> set of tetrahedron which are
> described by the indices to the data point vectors (x,y,z).
>
> A fourth optional argument, which must be a string or cell array of strings,
> contains extra options passed to the underlying qhull command. See the
> documentation for the Qhull library for details.
>
>
>
> **See also:** delaunay, delaunayn.

— Function File: T = **delaunayn** (P)  
— Function File: T = **delaunayn** (P, opt)  
> Form the Delaunay triangulation for a set of points.
> The Delaunay triangulation is a tessellation of the convex hull of the
> points such that no n-sphere defined by the n-triangles contains
> any other points from the set.
> The input matrix P of size `[n, dim]` contains n
> points in a space of dimension dim. The return matrix T has the
> size `[m, dim+1]`. It contains for each row a set of indices to
> the points, which describes a simplex of dimension dim. For example,
> a 2d simplex is a triangle and 3d simplex is a tetrahedron.
>
> Extra options for the underlying Qhull command can be specified by the
> second argument. This argument is a cell array of strings. The default
> options depend on the dimension of the input:
>
> - 2D and 3D: opt = `{"Qt", "Qbb", "Qc"}`- 4D and higher: opt = `{"Qt", "Qbb", "Qc", "Qz"}`
>
> If opt is [], then the default arguments are used. If opt
> is `{""}`, then none of the default arguments are used by Qhull.
> See the Qhull documentation for the available options.
>
> All options can also be specified as single string, for example
> `"Qt Qbb Qc Qz"`.

An example of a Delaunay triangulation of a set of points is

```
     rand ("state", 2);
     x = rand (10, 1);
     y = rand (10, 1);
     T = delaunay (x, y);
     X = [ x(T(:,1)); x(T(:,2)); x(T(:,3)); x(T(:,1)) ];
     Y = [ y(T(:,1)); y(T(:,2)); y(T(:,3)); y(T(:,1)) ];
     axis ([0, 1, 0, 1]);
     plot(X, Y, "b", x, y, "r*");
```

- Plotting the Triangulation- Identifying points in Triangulation
